# Transsynaptic Long-Term Potentiation in the Hippocampus of Behaving Mice

**DOI:** 10.3389/fnsyn.2021.811806

**Published:** 2022-01-20

**Authors:** Maria Teresa Romero-Barragán, Agnes Gruart, José M. Delgado-García

**Affiliations:** Division of Neurosciences, Pablo de Olavide University, Seville, Spain

**Keywords:** hippocampal synapses, long-term potentiation, high-frequency stimulation, mice, non-directly evoked long-term potentiation, input/output curves, paired-pulse facilitation

## Abstract

Long-term potentiation (LTP) is an experimental procedure that shares certain mechanisms with neuronal learning and memory processes and represents a well-known example of synaptic plasticity. LTP consists of an increase of the synaptic response to a control stimulus following the presentation of a high-frequency stimulation (HFS) train to an afferent pathway. This technique is studied mostly in the hippocampus due to the latter’s high susceptibility and its laminar nature which facilitates the location of defined synapses. Although most preceding studies have been performed *in vitro*, we have developed an experimental approach to carry out these experiments in alert behaving animals. The main goal of this study was to confirm the existence of synaptic changes in strength in synapses that are post-synaptic to the one presented with the HFS. We recorded field excitatory post-synaptic potentials (fEPSPs) evoked in five hippocampal synapses, from both hemispheres, of adult male mice. HFS was presented to the perforant pathway (PP). We characterized input/output curves, paired-pulse stimulation, and LTP of these synapses. We also performed depth-profile recordings to determine differences in fEPSP latencies. Collected data indicate that the five selected synapses have similar basic electrophysiological properties, a fact that enables an easier comparison of LTP characteristics. Importantly, we observed the presence of significant LTP in the contralateral CA1 (cCA1) area following the control stimulation of non-HFS-activated pathways. These results indicate that LTP appears as a physiological process present in synapses located far away from the HFS-stimulated afferent pathway.

## Introduction

LTP of synaptic strength is an experimentally induced technique commonly used for studying the mechanisms involved in learning and memory processes, since changes in synaptic efficacy of both events share many properties with respect to the underlying physiological, cellular, and molecular phenomena (Bliss and Collingridge, 1993; Gruart et al., 2006). LTP is usually induced in excitatory synapses of the hippocampus and other cortical and subcortical sites by the HFS of an afferent pathway to a given set of postsynaptic neurons and consists of the increase in the amplitude or slope of excitatory post-synaptic potentials (EPSPs) recorded intracellularly or in the surroundings of the postsynaptic cell as fEPSPs. This electrophysiological potentiation of postsynaptic responses can last from several minutes or hours during *in vitro* experiments (Bortolotto et al., 2001; Lynch, 2004) to days and weeks when synaptic electrophysiological events are recorded in alert behaving mammals (Abraham, 2003; Madroñal et al., 2007; Abraham and Williams, 2008).

Although there are many different ways of evoking and recording long-term changes in synaptic strength and functional efficacy, we are particularly interested here in the putative transsynaptic spread of LTP across relatively distant synapses (Helme-Guizon et al., 1998; Krug et al., 2001; Taylor et al., 2016). The question is whether LTP could be a general mechanism to activate distant but interconnected brain circuits involved in the different sensorimotor or cognitive aspects of newly acquired behavioral or mental abilities (Fernández-Ruiz et al., 2012).

The hippocampus seems to be a good place for the experimental approach to the above question. Indeed, the LTP of synaptic strength was described for the first time in the hippocampal formation (Bliss and Lømo, 1973) and it has been repeatedly studied since then with many different experimental procedures. The well-defined intrinsic circuit of the hippocampus and its specific ultrastructural organization in layers (Witter et al., 2000; Shepherd, 2004) have allowed an extensive and detailed study of LTP properties and putative functions (Collingridge et al., 1992; Bortolotto and Collingridge, 1993; Matsuzaki et al., 2004; Gruart et al., 2015; Volianskis et al., 2015; Korte and Schmitz, 2016; Fassin et al., 2020). However, most of these earlier studies describe only the effects of LTP in synaptic sites in direct contact (i.e., monosynaptic) with an activated afferent pathway, leaving further transsynaptic sites completely unexplored. *In vitro* studies present obvious technical limitations to the performance of transsynaptic recordings (Bortolotto et al., 2001; Volianskis and Jensen, 2003), but *in vivo* recordings carried out in alert behaving animals could certainly facilitate the proper study of LTP spread across cortical circuits following experimental (e.g., HFS-evoked) or functional (i.e., during actual learning) processes. For example, there are already studies carried out in behaving rats suggesting the presence of experimentally evoked transsynaptic LTP in hippocampal-to-contralateral medial prefrontal cortex (Taylor et al., 2016) and entorhinal cortex-to-contralateral dentate nucleus (Krug et al., 2001) circuits.

In this study, we aimed to explore any transsynaptic LTP in the hippocampal circuit in alert behaving mice. For this, we investigated the basic electrophysiological properties of five synapses of the hippocampal circuit (PP-CA3, PP-CA1, PP-cCA1, CA3-CA1, and CA3-cCA1) by performing input/output curves and testing paired-pulse facilitation. In a second step we studied changes in synaptic plasticity synapses directly (PP-CA3, PP-CA1) and non-directly (PP-cCA1, CA3-CA1, and CA3-cCA1) activated by HFS trains presented to the PP.

## Materials and Methods

### Experimental Animals

C57Bl/6 male adult mice (3–4 months old) obtained from the University of Granada Animal House (Granada, Spain) were used in this study. Animals were housed in standard cages (*n* = 5 per cage) with a covering grid and enrichment materials inside (cardboard rolls and paper). After surgery, animals were kept in individual cages for a better preservation of the implanted electrodes. The mouse room remained on 12 h light/dark periods, at constant room temperature (21.5 ± 1°C) and humidity (55 ± 8%). Food and water were provided *ad libitum*. Experimental procedures were carried out in accordance with European Union guidelines (2003/65/CE) and Spanish regulations (BOE 252/34367–91, 2005) for the use of laboratory animals in acute and chronic studies. All protocols were also approved by the Pablo de Olavide Ethics Committee (JA 06/03/2018/025). Only animals with electrodes implanted in the proper site and presenting correct fEPSP waveforms across the whole experiment were further considered in this study.

### Surgery

Animals were anesthetized with 1–2% isoflurane in a gas chamber provided with a calibrated Fluotec 5 (Fluotec-Ohmeda, Tewksbury, MA, United States) vaporizer, at a flow rate of 1–2 L/min oxygen (AstraZeneca, Madrid, Spain). Afterward, they were placed in a stereotaxic frame with a continuous supply of anesthesia (0.5% isoflurane) delivered by a special mouse mask (David Kopf Instruments, Tujunga, CA). Animals were covered with an electric blanket in order to maintain body temperature at 37°C.

**FIGURE 1:**
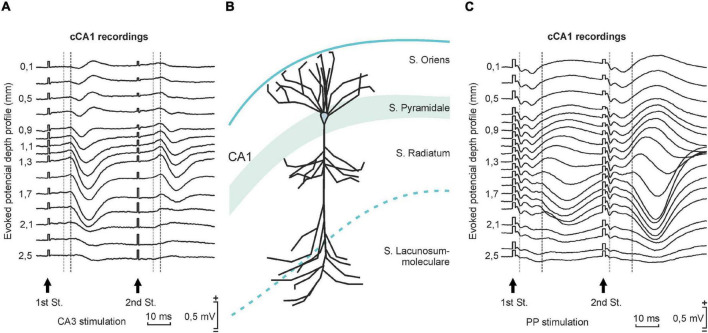
Selective examples of preliminary depth-profile recordings that help to characterize the fEPSPs evoked at the contralateral CA1 synapses included in this study. **(A)** Depth-profile representation of fEPSPs evoked in the contralateral CA1 area by paired-pulse stimulation (40 ms of interpulse interval, red arrows) of Schaffer collaterals (CA3 stimulation). Stimulus intensity was set at 40% of the intensity (mA) necessary to evoke maximum fEPSP responses at 1.3 mm depth. Recordings were carried out in steps of 100 and 200 μm. Note that the latency of the fEPSP (dark gray) is longer than the one for the homolateral CA3-CA1 synapse, i.e., stimulating and recording in the same hemisphere (light gray; obtained from Gruart et al., 2015). **(B)** A diagrammatic representation of the location of somas and dendrites corresponding to CA1 pyramidal cells and to dentate granule neurons. The different strata are indicated. **(C)** Another field-potential depth profile corresponding to fEPSPs evoked in the contralateral CA1 area by the paired-pulse stimulation of the ipsilateral perforant pathway.

First of all, depth profile recordings were carried out in anesthetized mice in order to characterize cCA1 responses to PP and CA3 stimulations (Figure 1) and to compare the collected fEPSPs with those evoked in the ipsilateral CA1 region (Gruart et al., 2015). For this purpose, a craniotomy was performed 2,2 mm posterior and 1,2 mm lateral to bregma. Following stereotaxic coordinates (Franklin and Paxinos, 2007) a stimulating electrode was implanted in the hippocampal CA3 region (A-P: –1.5 mm, L: –1.7 mm, and D: –1.5 mm) and in the perforant pathway (A-P: –3.8 mm and L: –2.0 mm relative to bregma, and 1.0 mm deep from the brain surface). A 10-μm glass micropipette filled with 2 M NaCl and held by a micromanipulator (Narishige, Tokyo, Japan) was used to record in 100 μm steps along the vertical axis of the cCA1 pyramidal layer.

**FIGURE 2:**
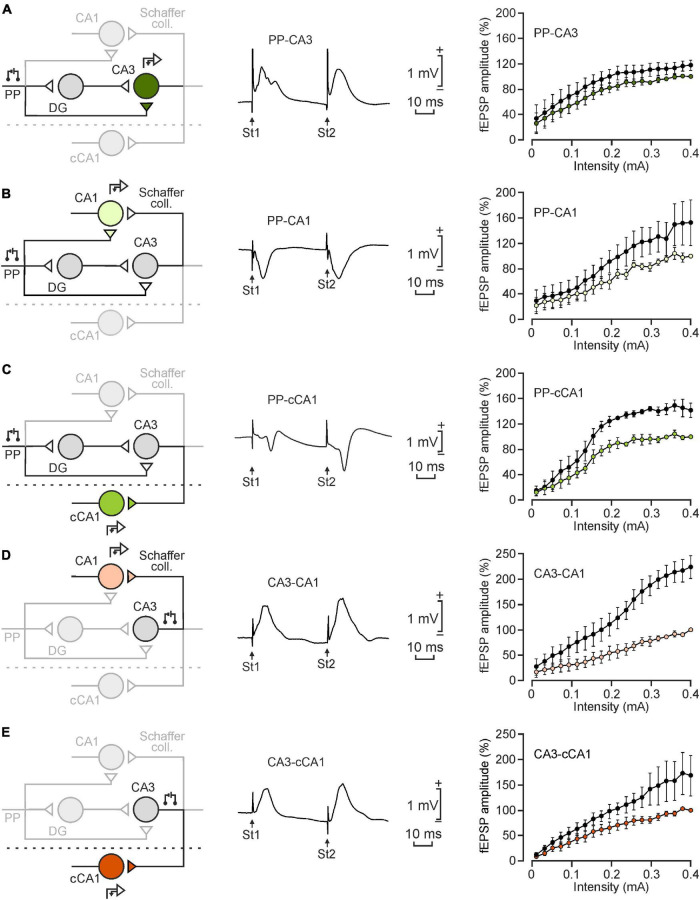
Input/output curves of the five selected synapses. **(A–E)** From left to right are illustrated a schematic representation of the synapse under study, a representative example of the fEPSP recording at 2 × Threshold intensities (St1: first stimulus; St2: second stimulus) and input/output curves collected from the five illustrated synapses: **(A)**, PP-CA3; **(B)**, PP-CA1; **(C)**, PP-cCA1; **(D)**, CA3-CA1; and **(E)**, CA3-cCA1. Computed curves were normalized as the percentage of amplitude values reached under the maximum intensity. Black circles stand for the second stimulus. Data were collected from 5 animals/synapse.

**FIGURE 3:**
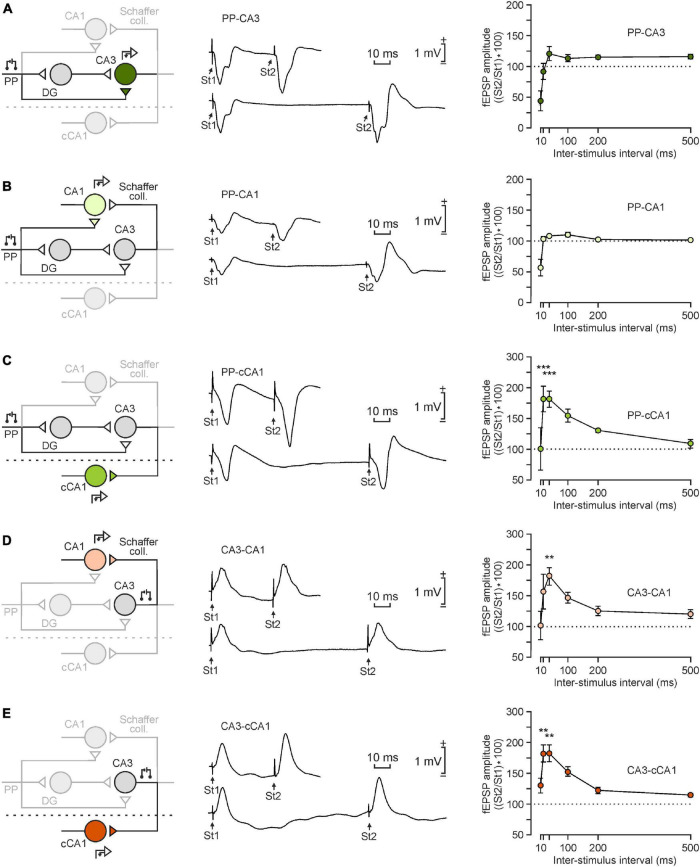
Paired-pulse facilitation of the five selected synapses. **(A–E)** From left to right are illustrated a schematic representation of the synapse under study, a representative example of fEPSP evoked by the paired-pulse stimulation (St1: first stimulus; St2: second stimulus) at 40 and 100 ms interstimulus interval, and the paired-pulse facilitation induced in the five illustrated synapses: **(A)**, PP-CA3; **(B)**, PP-CA1; **(C)**, PP-cCA1; **(D)**, CA3-CA1; and **(E)**, CA3-cCA1. Each synapse is represented by a different color. Black circles stand for the second stimulus. Data were collected from 4 animals/synapse. Every point for each animal represents the mean value of 10 stimuli (with its corresponding SEM). The five synapses presented similar facilitation patterns in the same interstimulus intervals [*F*_(5, 15)_ = 11.458; *P* < 0.001]. **P < 0.01, ***P < 0.001 in comparison with the values collected at 500 ms —i.e., the most similar to baseline values.

**FIGURE 4:**
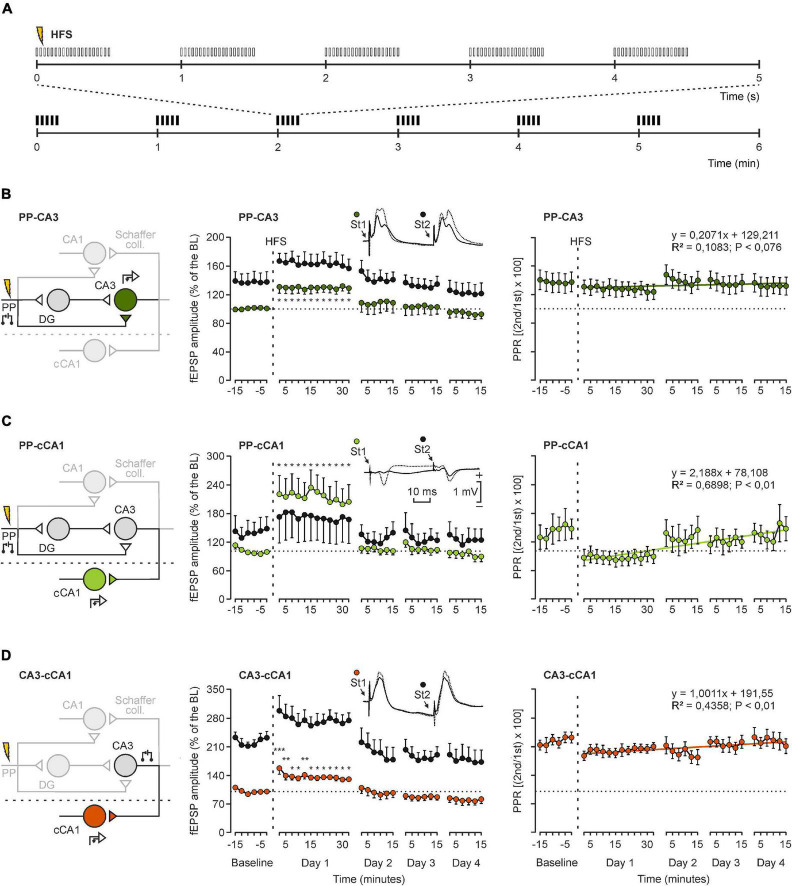
High-frequency stimulation of the PP evoked LTP in CA3 and cCA1. **(A)** The high-frequency stimulation (HFS) protocol consisted of 5 trains (200 Hz, 100 ms) of pulses at a rate of 1/s. These trains were presented 6 times in total, at intervals of 1/min. **(B–D)** Schematic representation of the stimulating and recording sites during the experiment (left), LTP evolution of PP-CA3 **(B)**, PP-cCA1 **(C)** and CA3-cCA1 **(D)** synapses (middle) and their paired-pulse ratios and linear regression lines (right). Insets illustrate representative examples of recorded fEPSPs from the three indicated synapses –not necessarily from the same mouse. Each circle represents the average amplitude computed from 15 stimulus presentations ± SEM. LTP was evoked in the 3 synapses by HFS of the PP. In order to obtain a baseline, animals were stimulated every 10 s for 15 min in the afferent pathway. After HFS, the same stimulus was presented at the initial rate (6/min) for another 30 min. Recording sessions were repeated on 3 additional days (15 min each) (*n* = 10). *P < 0.05; **P < 0.01; ***P < 0.001 (one-way ANOVA, contrast analysis against baseline values).

**FIGURE 5:**
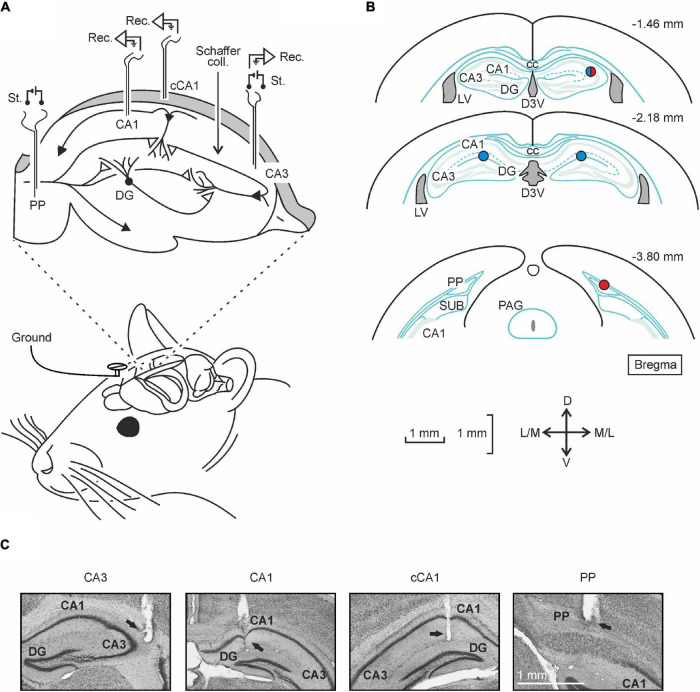
Experimental design. **(A)** Mice were implanted with bipolar stimulating (St.) electrodes in the perforant pathway (PP) and the hippocampal CA3 area, and with recording (Rec.) electrodes in the hippocampal CA1 areas of both hemispheres. A bare silver wire was affixed to the occipital bone as a ground. **(B)** A diagrammatic representation of three coronal slices of mouse brain (Franklin and Paxinos, 2007) illustrating stimulation (red) and recording (blue) sites. **(C)** Representative micrographs illustrating the final location of stimulating and recording electrodes indicated with a black arrow. Scale bar is indicated at the bottom right. D, L, M, and V stand for dorsal, lateral, medial, and ventral, respectively; PP, perforant pathway; DG, dentate gyrus; Schaffer coll, Schaffer collaterals; CA3, *cornu ammonis*-3; CA1, homolateral *cornu ammonis*-1; and cCA1, contralateral *cornu ammonis*-1.

For chronic recordings (Figures 2–4), animals were implanted with two bipolar stimulating and two recording electrodes. Stimulating electrodes were implanted in the perforant pathway and the hippocampal CA3 region using the stereotaxic coordinates already mentioned in the previous paragraph. The latter also worked as a recording electrode when needed. Recording electrodes were implanted in the CA1 area of both hemispheres (A-P: –2.2 mm, L: –1.2 mm, and 1.2 mm, and D: –1.3 mm; Figure 5). This experimental design enabled the study of five hippocampal synapses: PP-CA3, PP-CA1, PP-cCA1, CA3-CA1, and CA3-cCA1 (Figures 2–4). Animals used in the LTP experiment lacked the recording electrode in iCA1 to avoid any unnecessary brain damage. All electrodes were made from 50 μm, Teflon-coated tungsten wire (Advent Research, Eynsham, United Kingdom), with 0.5 mm of their tips bared. Two screws, affixed to the frontal and interparietal bones and soldered to bare silver wires, served as ground. Wires were soldered to two (6-pin and 4-pin) sockets (RS Amidata, Madrid, Spain) and covered with dental cement in order to secure the implanted electrodes. Further details about these experimental procedures can be found elsewhere (Gruart et al., 2006).

**FIGURE 6:**
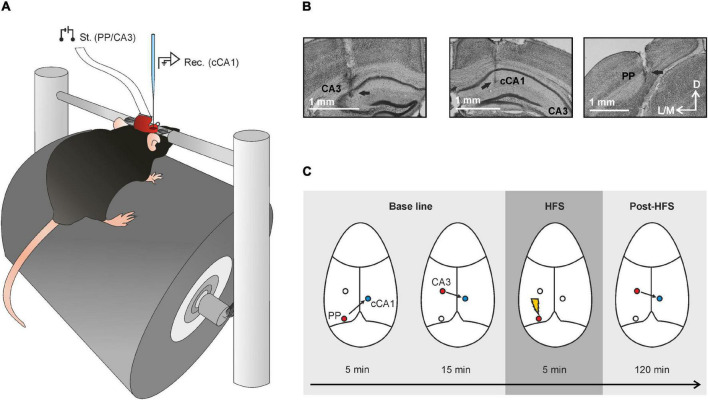
Experimental procedures for recordings in alert head-fixed mice. **(A)** Mice were placed on a head-fixed set-up for the recording of cCA1 potentials evoked by stimulation of either the PP or the hippocampal CA3 area. **(B)** Photomicrographs of CA3, cCA1, and PP showing the electrode scar. **(C)** Schematic representation of the involved synapses during the experiment with indication of the stimulating (red) and recording (blue) locations. A 5-min baseline was recorded for the PP-CA3 synapse to ensure that the position of the electrode in the PP was good enough for the successive HFS, followed by a 15-min baseline for the CA3-cCA1 synapse. After the 5-min HFS, post-HFS recordings were carried out in the CA3-cCA1 synapse for 120 min.

**FIGURE 7:**
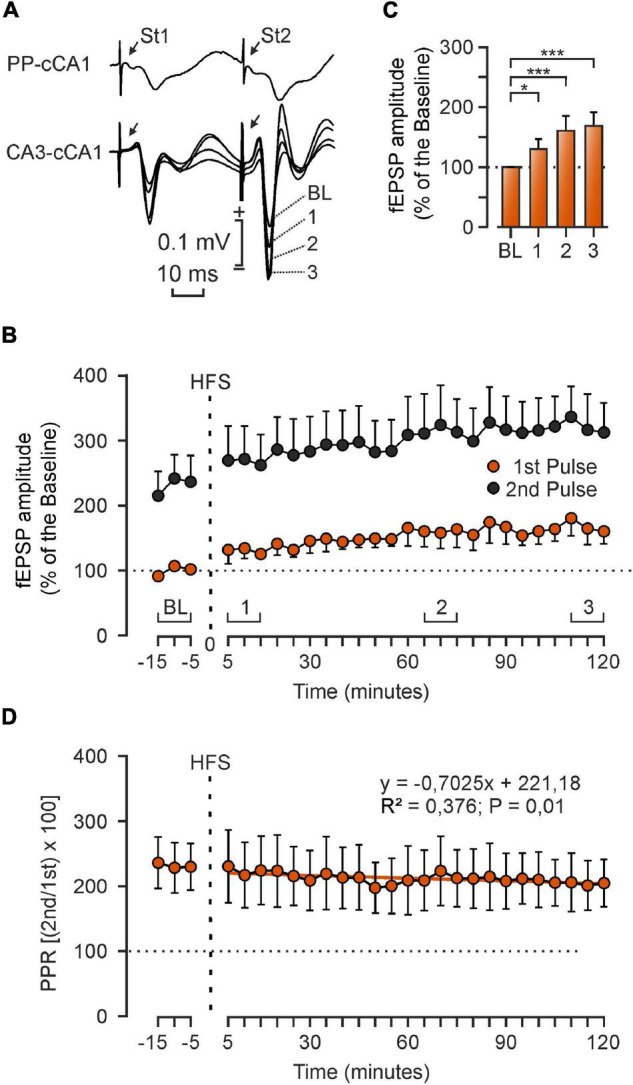
Transsynaptic effects of LTP in alert head-fixed mouse. **(A)** Representative fEPSP collected from the PP-cCA1 and CA3-cCA1 synapses. **(B)** After presenting an HFS protocol to the ipsilateral PP, the LTP effects evoked in the CA3-cCA1 synapse persisted for at least 2 h. For the baseline, animals were stimulated every 20 s for 15 min in the CA3 area. The same stimuli were applied in the post-HFS recordings, which lasted 120 min. **(C)** Mean ± SEM. 1st pulse fEPSP values 1–15 min of baseline (BL), and 1–15 min (1), 65–75 min (2) and 110–120 min (3) after the HFS. Data were collected from 5 animals. *P < 0.05, ***P < 0.001 (one-way ANOVA). **(D)** The relationship between 2nd and 1st pulses—the paired-pulse ratio (PPR) calculated with the formula (2nd/1st × 100)—presents a linear regression line with a negative slope. Equations corresponding to the linear regression analyses of PPRs are indicated in the plot.

For the head-fixed experiment (Figures 6, 7), the skull was drilled at the previously mentioned coordinates for PP, CA3, and cCA1. Stimulating electrodes were implanted in PP and CA3 and soldered to a 4-pin socket (RS Amidata, Madrid, Spain). In addition, a recording chamber was built with dental cement around a craniotomy performed over the cCA1 area. The bone window was covered with gauze and bone wax until the recording session. In this case, mice were also implanted with a small metallic head-plate which would keep the animal’s head attached to the recording setup in a stable position during experimental sessions. The holding plate and the implanted electrodes were affixed to the skull with the help of two small screws covered with cyanoacrylate and dental cement. A bare silver wire was soldered to the screws, serving as ground, and had a small loop at the other end that was left uncovered by the cement (López-Ramos et al., 2018).

After surgery, mice rested for a week for recovery until experiments began.

### Stimulation and Recording Procedures

Chronic recordings were performed in 6 animals at a time, which were introduced into individual, small (5 cm × 5 cm × 10 cm) see-through plastic chambers located in a larger Faraday box (30 cm × 30 cm × 20 cm). fEPSPs were evoked by electrical pulses delivered by Cibertec CS20 stimulators across ISU-220 isolation units (Cibertec, Madrid, Spain) and recorded with Dagan Corporation EX4-400 Quad Differential amplifiers (Dagan Corporation, Minneapolis MN United States) at a bandwidth of 0.1 Hz–10 kHz, through a high-impedance probe (2 × 1,012 Ω, 10 pF).

Input/output curves (Figure 2) of each of the five selected synapses were tested by applying 100-μs, square, biphasic paired pulses (40 ms of interstimulus interval) of increasing intensities (0.02–0.4 mA, in steps of 0.02 mA). Ten pulses of each intensity were presented to the animals at intervals of > 10 s to avoid stimulus interactions (Gruart et al., 2006).

For the paired-pulse facilitation test (Figure 3) mice were stimulated in each synapse with paired pulses of increasing interstimulus interval (10, 20, 40, 100, 200, and 500 ms; ten pulses each) with intensities corresponding to 40% of the intensity necessary to evoke a saturating fEPSP response (Gureviciene et al., 2004).

In order to induce LTP in the chronic experiments (Figure 4), we applied a high-frequency stimulation (HFS) protocol always in the PP, consisting of five 200-Hz, 100-ms trains of pulses at a rate of 1/s. This protocol was presented 6 times (at 1/min). A 15-min baseline was established before LTP induction by evoking fEPSPs with paired (40-ms interstimulus interval) 100-μs square, biphasic pulses at a rate of 6/min. Pulse intensity was set at 40% of the amount necessary to evoke a maximum fEPSP response (0.05–0.4 mA; Gureviciene et al., 2004; Gruart et al., 2006). In order to prevent the appearance of large amplitude population spikes and/or hippocampal seizures, intensities used for evoking baseline recordings were maintained for the HFS protocol. None of the animals of this study suffered any of those unwanted events after receiving the HFS protocol, as checked in the on-line EEG recordings. Post-HFS recordings were acquired at the same intensity and frequency as in the baseline for 30 min the first day and for 15 min the 3 following days. fEPSPs were averaged every 15 pulses in order to measure the amplitude in both baseline and post-HFS recording and represented as a percentage of baseline (100%) values. With the purpose of recording the five hippocampal synapses, the following experimental design was followed: first, fEPSP were evoked in CA3, CA1, and cCA1 by the stimulation of the PP. Second, fEPSP were evoked in CA1 and cCA1 by stimulating the CA3 area. These two configurations were alternated in a way that did not affect fEPSP recordings.

For the head-fixed recordings, mice were individually placed on a foam-covered running wheel supported by a system of aluminum bars. The animal had freedom of leg movements, but its head plate was screwed to the two tightened horizontal holding bars (Figure 6A). Further information about this recording device can be found in a previous study by some of us (Heiney et al., 2014; López-Ramos et al., 2018). Mice had 3 days of habituation (5, 15, 25 min) in order to minimize stress during recording sessions. fEPSPs were evoked with the above-described stimulation equipment and recorded with an AC/DC differential amplifier (Model 3000, A-M system, Carlsborg, WA) at a bandwidth of 0.1 Hz–3 kHz, through a high-impedance probe (2 × 1,012 Ω, 10 pF). Recordings in the contralateral CA1 area (cCA1) were carried out with a 10-μm glass micropipette filled with 2 M NaCl and mounted on a micromanipulator (M-2, Narishige, Tokyo, Japan). The electrode was introduced into the brain and the correct waveform searched for, using as a guide the depth-profile fEPSPs illustrated in Figure 1, obtained by stimulating with 40-ms ISI pulses in either PP or CA3. Firstly, a 5-min baseline was established in the PP-cCA1 synapse looking for a good fEPSP waveform to ensure that the position of the PP stimulating electrode was appropriate for a correct LTP induction. Next, a 15-min baseline for the CA3-cCA1 synapse was recorded. After that, LTP induction was elicited by presenting the above-described HFS protocol. Post-induction effects were recorded for 2 h. Electrical stimulation during baselines and post-HFS recordings consisted of 100-μs, square, biphasic, paired pulses (40 ms of interstimulus interval) at a rate of 1/20 s. Data were analyzed to measure fEPSP amplitudes, which were represented as a percentage of the baseline (100%) values. For that, fEPSPs were averaged (*n* = 15) every 5 min. The same stimulating and recording instruments were used for the depth-profile recordings. Stimulation intensity was set to obtain the 40% of the maximum response at 1,3 mm depth and maintained for the rest of the experiment.

### Histology

At the end of the experiments, mice were deeply anesthetized (sodium pentobarbital, 50 mg/kg) and perfused transcardially with 0.9% saline, followed by 4% paraformaldehyde phosphate buffer (PFA) as a fixative. Their brains were removed and maintained in the same solution overnight at 4°C. After a cryoprotectant treatment with an increasing gradient of sucrose in PBS (5, 15, and 30%), brains were cut into 50-μm slices in a cryotome (Leica, Wetzlar, Germany) and the slices of interest were mounted on gelatinized glass slides and stained with 0.1% toluidine blue (Nissl technique). Photomicrographs of electrode scars were taken using a 5 × objective of a Leica DMRE microscope equipped with a Leica DFC550 camera, and with the help of the LAS V4.2 software (Leica Microsystems GmbH, Wetzlar, Germany).

### Data Collection and Analysis

fEPSPs and 1V square pulses corresponding to stimulus presentations were digitally stored on a computer through the analog/digital converter CED 1401 Plus (CED, Cambridge, England), at a sampling frequency of 5 kHz, and with an amplitude resolution of 16 bits. fEPSP amplitudes were quantified off-line using CED Spike 2 and Signal (Systat Software, San Jose, CA, United States) programs. For this, we averaged the amplitude of 15 fEPSPs collected every 2.5 min (chronic recordings) or 5 min (acute recordings). We used Microsoft Excel (Microsoft, Redmond, WA, United States) and CorelDraw (Corel Corporation, Ottawa, Canada) programs for data representation and the Sigma Stat for Windows package for statistical analysis. Unless otherwise indicated, data are represented as the mean ± SEM. Acquired data from PPF were analyzed using a two-way ANOVA, with synapse and interval as factors. LTP data were analyzed using a one-way ANOVA test, with sessions as repeated measure. When a normality test failed, the significance (*p*-value) was calculated with the Friedman Repeated Measures Analysis of Variance on Ranks test, a non-parametric method. Contrast analysis was added for a further study of significant differences after parametric (Student-Newman-Keuls Method) and non-parametric (Tukey) tests. In the PPF study, values of the 500 ms interstimulus interval were considered the baseline for contrast analysis.

## Results

### The Five Selected Hippocampal Synapses Share Similar Basic Electrophysiological Properties

As explained in detail in “Materials and Methods” section, animals (*n* ≥ 5 animals/synapse) were prepared for different electrophysiological studies in five hippocampal synapses: PP-CA3, PP-CA1, PP-cCA1, CA3-CA1, and CA3-cCA1 (see Figures 5A,B). The proper electrode position was determined during surgery with the help of the field-potential depth profile recordings obtained in previous experiments involving ipsilateral (Gruart et al., 2015) and contralateral pathways (Figure 1). Once the experiments ended, we checked the final location of the implanted electrodes by means of histological techniques (Figure 5C).

In a first series of experiments on alert behaving mice, we studied the basic properties of the five synapses. For input/output curves, mice were presented with paired pulses (40 ms of interpulse interval) of increasing intensities (0.02–0.4 mA, in steps of 0.02 mA). All synapses presented similar input/output curves, with a sigmoid-like shape, to the presentation of the first pulse (Figure 2, right column).

We paid particular attention to the relationships between fEPSPs evoked by the second pulse and those evoked by the first one. Second pulse presentation evoked responses with a higher facilitation in the five selected synapses as the intensity became higher, especially in the CA3-CA1 synapse (Figure 2D). In contrast, the CA3-cCA1 synapse presented lower facilitation values at high stimulus intensities (Figure 2E). Interestingly, and as already indicated in a previous study (Gruart et al., 2015), the ratio 2nd/1st pulse in the PP-CA3 synapse stayed more stable across the increasing stimulus intensities with no signs of paired-pulse facilitation across the whole range of stimulus intensities (Figure 2A).

The additional purpose of the input/output curves test was to set the appropriate stimulation intensity for the rest of the experiments in each mouse—i.e., the intensity able to evoke an fEPSP with about 40% of the maximum amplitude obtained in the input/output test. Recorded fEPSP waveforms (Figure 2, middle panel) were indicative of the position of the electrode tip relative to the neuron’s position in the hippocampal intrinsic circuit—namely, closer to the apical dendrites (negative) or to the somas (positive). All synapses presented evoked fEPSPs with latencies in the range of 1.5–3.5 ms, except for the PP-cCA1 synapse, which presented latencies ranging from 5.5 to 8 ms. This difference clearly indicates that this pathway is not monosynaptic, as illustrated in Figure 2C (left).

We also performed a paired-pulse facilitation test (*n* ≥ 4 animals/synapse) at increasing (10, 20, 40, 100, 200, and 500 ms) interstimulus intervals. This experimental procedure offered an idea of the changes in short-term plasticity in the five synapses included in this study. Indeed, it is generally accepted that changes in paired-pulse facilitation (or depression) are related to the probability of release of the neurotransmitter at the presynaptic terminal (Volianskis and Jensen, 2003; Lauri et al., 2007).

The five selected synapses illustrated in Figure 3 presented statistically significant differences after a 2-way ANOVA analysis on the effect of interstimulus interval [*F*_(5, 15)_ = 11.458; *P* < 0.001] and synapse [*F*_(4, 12)_ = 10.520; *P* < 0.001], as well as their interaction [*F*_(20, 60)_ = 3,016; *P* < 0.001]. Nevertheless, a deeper observation of the differences between synapses showed that there were two separated patterns. Firstly, synapses PP-CA3 (Figure 3A) and PP-CA1 (Figure 3B) presented similar responses since there were no significant differences (*P* = 0,756) between them. And, secondly, the other three hippocampal synapses (Figures 3C,D) did not present significant differences among them (*P* ≥ 0.878), either.

Regarding the comparisons on the intervals effect, the 40-ms interstimulus interval presented the highest paired-pulse facilitation [*t*_(3, 0_._05)_ = 4.158; *P* = 0.01]. However, a further analysis with all Pairwise Multiple Comparison Procedures (Holm-Sidak method) determined that not all of the synapses present the same magnitude of facilitation. Thus, the paired-pulse facilitation in the 40-ms interval in the PP-CA3 and PP-CA1 synapses was significantly lower than in CA3-CA1, CA3-cCA1, and PP-cCA1 (*P* < 0.01). The two synapses involving the contralateral CA1 area (PP-cCA1 and CA3-cCA1) also presented a statistically significant facilitation at the 20-ms interpulse interval (*P* < 0.01; Figures 3C,E).

### The High-Frequency Stimulation of the Perforant Pathway Evokes Long-Term Potentiation Not Only in the Different Ipsilateral Synapses but Also in the Contralateral CA1 Area

In order to study the functional changes evoked in the hippocampal synapses PP-CA3, PP-cCA1, and CA3-cCA1 following the experimental induction of LTP, 9–10 animals/synapse were presented with a well-known HFS protocol (Gruart et al., 2006), illustrated in Figure 4A. We focused on these main synapses involved in our hypothesis to avoid any unnecessary physical damage caused by electrodes implantation in the small mouse brain, thereby reducing alterations in synaptic transmission within the intrinsic hippocampal circuit. HFS trains were always presented at the same PP site (see left column of Figures 4B–D). Prior to the HFS protocol, a 15-min baseline recording was carried out in each synapse (at a rate of 6 stimuli/min). The stimulus intensity was always set at about 40% of the intensity necessary to evoke a maximum fEPSP response. Evoked fEPSPs were recorded for 1 h after the HFS induction at the indicated rate, and again during the next 3 days (30 min each), as previously explained in the “Materials and Methods” section.

We measured fEPSP amplitudes of the synapses PP-CA3, PP-cCA1, and CA3-cCA1 setting the cursor at the latencies of 2.6 ± 0.4, 6.1 ± 0.7, and 3.2 ± 0.5 ms after the artifact generated by the stimulus, respectively. LTP data are represented as a percentage of the baseline (100%) values and are shown in Figures 4B–D (right column). LTP effects recorded in the CA3 region after presenting the HFS protocol in the PP [χ^2^_(35, 315)_ = 155.254; *P* < 0.001] persisted for 2 days, reaching 130 ± 8.79% of baseline values, and gradually returned to basal values. When recording in the CA1 area of the contralateral hemisphere, there was also a significant increase of synaptic efficacy during the 1st day after the LTP induction, but this increase in synaptic strength returned to basal values the second day. The PP-cCA1 synapse presented a potentiation of 233.1 ± 37.9% [χ^2^_(35, 315)_ = 157.695; *P* < 0.001], and CA3-cCA1 values rose to 159.64 ± 14.00% of the baseline [*F*_(35, 280)_ = 11,355; *P* < 0.001]. Intriguingly, the latter synapse presented a slight depression in evoked fEPSP amplitudes the next 3 days of recordings. We also calculated the paired-pulse ratio (PPR) following LTP induction as an indicative of the relationship between the 1st and the 2nd pulse and, hence, the possibility of changes in presynaptic components of these hippocampal synapses. The PP-cCA1 synapse presented the steepest regression line (slope: 2,188), followed by the CA3-cCA1 synapse (slope: 1.0011). The PP-CA3 synapse presented PPRs across LTP sessions with minimum changes in slope (slope: 0.207). These results indicated that LTP can be evoked in hippocampal circuits not only in the presynaptically stimulated synapse, but also in synapses transsynaptically distant from the stimulated afferent pathway.

### Long-Term Potentiation Evoked at the CA3-cCA1 Synapse After Presenting High-Frequency Stimulation Trains in the Perforant Pathway in Head-Fixed Mice

In order to confirm the transsynaptic effects of the LTP evoked in the hippocampal intrinsic circuit, we tried a different approach. In this case, animals were surgically prepared for being placed in a head-holding system that enabled recording fEPSPs using a 10-μm tip glass micropipette for cCA1 recordings (Figure 6A). This recording system allows a more accurate position of the electrodes while the animal’s head is fixed to the set up and with no need of anesthesia, avoiding unwanted effects on NMDA receptors. LTP was evoked in 5 animals by presenting a HFS protocol in PP to study changes in synaptic strength of the CA3-cCA1 synapse. The final location of electrodes and glass pipette was also confirmed by histological procedures (Figure 6B). The experimental procedure illustrated in Figure 6C was as follows. An initial baseline was determined to establish the correct position of the stimulating electrode in the PP, as confirmed when recording fEPSP profiles in the cCA1 area (illustrated at the top of Figure 6A). After that, a second fEPSP baseline, this time in the CA3-cCA1 synapse, was recorded for 15 min at a rate of 3/min before presenting the HFS protocol in the PP. Changes in the synaptic efficacy were recorded for 2 h at the same initial rate after the LTP induction (Figure 7A bottom). Interestingly, again we noted an increase in the synaptic efficacy of the CA3-cCA1 synapse that lasted for at least 2 h [*F*_(8, 32)_ = 5.920; *P* < 0.001; Figure 7B]. In this case, unlike the regression line of the PPR presented a negative slope (–0.7025). It is not surprising that these results differ from those collected from the chronic experiments in the same synapse, given that in this case recordings are from only one recording session.

As far as we know, there is no report indicating the presence in mice of any noticeable direct axonal projection from the entorhinal cortex (via the PP) to the dorsal CA1 area of the contralateral hemisphere (van Groen et al., 2003; Sun et al., 2018; Tao et al., 2021). Thus, the present results strongly suggest that a significant increase in synaptic strength is transsynaptically evoked by LTP induction in the PP-cCA1 synapse.

## Discussion

### Long-Term Potentiation Could Be a Physiological Process

The electrophysiological study presented here demonstrates that LTP experimentally induced by HFS in the PP transsynaptically propagates to the contralateral hippocampal CA1 area across the CA3-cCA1 projection. It is evidenced the longer latency of fEPSPs evoked in the cCA1 area by PP stimulation, suggestive of a disynaptic projection, and by the presence of LTP in the CA3-cCA1 synapse not evoked by previous direct HFS of the CA3 area. Even in the case that some entorhinal fibers crossing to the contralateral side were activated in these experiments this fact will not explain the presence of LTP at CA3-cCA1 synapses. These interesting results further support two seminal studies reporting the induction of LTP in the dentate gyrus by HFS stimulation of the contralateral PP (Krug et al., 2001) and the presence of LTP in the contralateral medial prefrontal cortex transsynaptically evoked by stimulation of the ipsilateral ventral hippocampus in behaving rats (Taylor et al., 2016). In addition, these results suggest that this transmission from an experimentally potentiated synapse to the contiguous one could happen by means of physiologically evoked action potentials, and strongly support classical contentions (Bliss and Collingridge, 1993; Citri and Malenka, 2008; Neves et al., 2008; Dringenberg, 2020) indicating that LTP is an actual functional mechanism that can be studied in true physiological conditions.

### Basic Electrophysiological Properties of the Five Selected Synapses

The latency of fEPSPs evoked at the studied synapses varied according to their physiological features. All of them but PP-cCA1 are monosynaptic projections in mice. The PP-cCA1 synapse presented the largest latency, since it is a disynaptic pathway consisting of the successive projection from entorhinal cortex axons to the CA3 region and its callosal projections to the cCA1 area (Finnerty and Jefferys, 1993). This is known to be a long-range projection (Yang and Sun, 2018), which is consistent with the larger latency observed in this study. Apparently, there is no significant direct axonal projection from the entorhinal cortex to the dorsal cCA1 area, as suggested in recently published works (van Groen et al., 2003; Sun et al., 2018; Tao et al., 2021). Consequently, for this study we focused on this disynaptic pathway to explore the transsynaptic effects of LTP. fEPSP waveforms recorded in these experiments presented positive as well as negative shapes. As illustrated in Figure 1, the final position of the recording electrode at the end of the implantation procedure explains the different profiles collected, depending on the location of the recording tip with respect to the sources or sinks of the selected synapses (Bliss and Lømo, 1973; Bliss and Collingridge, 1993; Gruart et al., 2006, 2015; Madroñal et al., 2009). Responses to PP stimulation (synapses PP-CA3, PP-CA1, and PP-cCA1) presented an earlier component corresponding to the direct connection we were interested in, and a later component evoked by the hippocampal trisynaptic circuit, which comprehends the DG. As known, the PP projects its axons to the granule cells of the DG and these send the information through the mossy fibers to the pyramidal cells of CA3.

The initial aim of this work was to study the basic properties of five different synapses belonging to the intrinsic hippocampal circuit in alert behaving mice by carrying out input/output curves and paired-pulse stimulation tests. The input/output curves of the five synapses presented sigmoid-like shapes, like the ones described in a previously published work (Gruart et al., 2015). For the selected synapses, peak fEPSP amplitudes evoked by the first pulse reached similar values, including those evoked at the CA3-cCA1 synapse, indicative of shared functional properties in this regard. With respect to the response of the selected synapses to paired-pulse stimulations at different time intervals, and in accord with previous reports in alert behaving mice (Gruart et al., 2006, 2015), the CA3-CA1 synapse presented the highest paired-pulse facilitation, indicating that this is a functionally weak projection that can be easily increased (Madroñal et al., 2009). Similar results were collected for CA3-cCA1 and PP-cCA1, indicating that this is a basic property of collateral Schaffer projections onto CA1 and cCA1 pyramidal cells. In contrast, PP-CA3 and PP-CA1 did not present signs of paired-pulse facilitation, indicating, according to the residual calcium hypothesis, the presence of a strong synaptic response to the first applied stimulus, leaving low amounts of residual calcium and hence reducing the probability of vesicle release for the presentation of the second pulse (Thomson, 2000; Zucker and Regehr, 2002). These minor differences in the magnitude of the facilitation/depression response could be of some relevance from a functional point of view within the intrinsic hippocampal circuit (Turrigiano and Nelson, 2004; Madroñal et al., 2009).

### Main Characteristics of Mono- and Disynaptically Evoked Long-Term Potentiation Responses

In a second step, we studied changes in synaptic efficacy of the three selected synapses after inducing LTP by HFS of the PP in alert behaving mice. The significant effects of the LTP induced in the PP were recorded not only in the hippocampal CA3 area, but also in the contralateral CA1 region. Then, as PP-cCA1 is a disynaptic pathway, we also checked the monosynaptic projection from CA3 to cCA1, finding that the synaptic efficacy was also increased at this synapse, which did not receive the HFS train directly. As above indicated, the LTP evoked at the CA3-cCA1 synapse cannot be explained by the putative presence of activated entorhinal fibers projecting to the contralateral CA1 area. As shown in Figures 4C,D, LTP decreased to baseline values after the first day in the projections involving cCA1. Thus, we decided to determine how long these effects lasted within the first session after the HFS was applied to the PP. For this, we further confirmed the transsynaptic effects of LTP evoked in head-fixed mice, applying the HFS protocol in the PP and recording the fEPSPs of the CA3-cCA1 synapse for up to 2 h. The rather short duration of this transsynaptically evoked LTP could be due to the fact that it was not associated with any actual learning acquired by the stimulated animal (Madroñal et al., 2009, 2016). This technique enabled a more accurate control of the electrode position, since it was an acute procedure, meaning that the glass micropipette was implanted in the same recording session. At the same time, the head-fixed set-up granted access to the unanesthetized mouse brain, without missing small polysynaptic activities usually unrecorded with chronically implanted metal electrodes.

The results presented here agree with those of a previous study carried out by Krug et al. (2001), in which the authors were very able of evoking an LTP in the dentate gyrus following the HFS stimulation of the contralateral PP; they proposed that this transsynaptic LTP was evoked by disynaptic potentials induced via the commissural fibers connecting the two entorhinal cortices. In a subsequent report, Taylor et al. (2016) were able to evoke LTP in the contralateral medial prefrontal cortex after inducing HFS of the vHPC; they ascribed this result to the successive activation of a disynaptic crossed pathway. Based on the use of HFS protocols with different frequencies, Yeckel and Berger (1998) proposed that the mono- (by the PP) or disynaptic (by mossy fiber afferents) induction of LTP in the CA3 area by HFS of the ipsilateral entorhinal cortex depends upon the selected stimulation pattern. These changes in synaptic strength caused by HFS in a non-directly afferent pathway represent a very interesting event that could shed some light on the way our brain processes learning and memory phenomena. In fact, this finding is consistent with the currently accepted idea that long-term memories do not remain in restricted and/or isolated regions of the brain (Lashley, 1950; Wiltgen et al., 2004; Josselyn and Tonegawa, 2020), but that they are stored and consolidated in wide cortical neural networks (McClelland et al., 1995; Frankland and Bontempi, 2005). It has also been proposed that plastic phenomena induced by experimental procedures may intensify spontaneous information flow across specific transsynaptic pathways (Smirnova et al., 1993; Davis et al., 1996); this could represent a putative network mechanism underlying types of memory involving multiple sensory-motor components and, obviously, multiple brain structures (Fernández-Ruiz et al., 2012). Nonetheless, we have shown here that LTP was transsynaptically evoked in the CA3-cCA1 pathway as a persistent consequence of the LTP evoked in the CA3 area by HFS of the ipsilateral PP—a finding not reported until now. In this regard, further attention should be paid to the still unknown properties of physiologically evoked LTP of cortical circuits. An appropriate approach for this would be to study LTP effects in other well-known cortical and subcortical excitatory synapses organized in cascade circuits and also involved in learning and memory processes.

## Data Availability Statement

The original contributions presented in the study are included in the article/supplementary material, further inquiries can be directed to the corresponding author/s.

## Ethics Statement

The animal study was reviewed and approved by the Pablo de Olavide Ethics Committee (JA 06/03/2018/025).

## Author Contributions

AG and JD-G conceived and designed the experiments and contributed to materials. MR-B performed the experiments. MR-B, AG, and JD-G designed the figures and analyzed the data. MR-B and JD-G wrote the manuscript. All authors revised the final version of the manuscript.

## Conflict of Interest

The authors declare that the research was conducted in the absence of any commercial or financial relationships that could be construed as a potential conflict of interest.

## Publisher’s Note

All claims expressed in this article are solely those of the authors and do not necessarily represent those of their affiliated organizations, or those of the publisher, the editors and the reviewers. Any product that may be evaluated in this article, or claim that may be made by its manufacturer, is not guaranteed or endorsed by the publisher.
